# A Radioresistant‐Tumor‐Targeted Nanoparticle for X‐Ray‐Controlled Nitric Oxide Release to Potentiate Radiotherapy

**DOI:** 10.1002/advs.202518233

**Published:** 2026-04-22

**Authors:** Wanze Zhang, Xiaoyan Yin, Ting Wang, Hongfu Zhao, Zhipeng Zhao, Jinbao Wang, Cheng Tao, Xuanchu Ge, Yanze Li, Linlin Liu, Fuxin Xue

**Affiliations:** ^1^ Department of Radiation Oncology China‐Japan Union Hospital of Jilin University Changchun China; ^2^ Department of Radiation Oncology National Cancer Center/National Clinical Research Center for Cancer/Cancer Hospital Chinese Academy of Medical Sciences and Peking Union Medical College Beijing China; ^3^ Department of Pathology China‐Japan Union Hospital of Jilin University Changchun China; ^4^ Department of Radiation Oncology Physics and Technology Shandong Cancer Hospital and Institute Shandong First Medical University and Shandong Academy of Medical Sciences Jinan China

**Keywords:** active targeting, biomaterials, controlled release, nitric oxide, radiation therapy

## Abstract

Nitric oxide (NO) treated radioresistant tumors by relieving hypoxia and blocking DNA repair, but its nonselective toxicity has precluded therapeutic use. Here, we introduce a radioresistant tumor‐selective NO nanogenerator that releases NO exclusively within the irradiated field. We identified BNN6 as a uniquely radiosensitive NO donor and loaded it into Glucose‐Regulated Protein 78 (GRP78)‐targeted nanocarrier to obtain PBTN, exploiting the overexpression of GRP78 in radioresistant cancers for selective accumulation. Upon irradiation, BNN6 undergoes one‐electron reduction to release NO exclusively within the irradiated volume. NO combines radiation‐induced reactive oxygen species to form peroxynitrite, provoking tumor DNA breaks while simultaneously suppressing DNA repair. In CT26 tumor‐bearing mice, the combination of radiotherapy with PBTN and anti‐PDL1 antibody achieved a tumor growth suppression of 96.5% and 80% survival at 40 days post‐treatment. This tumor‐targeted, irradiation‐triggered NO nanogenerator thus offers a safe, precise, and translatable strategy to overcome radioresistance.

## Graphical abstract

The PBTN targets GRP78 and, upon X‐ray irradiation, generates ONOO^−^ to induce immunogenic cell death, thereby activating CD8^+^ T cells and establishing a systemic antitumor immune response.

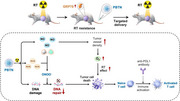

## 1 Introduction

Radiotherapy (RT) serves as a cornerstone for treating localized solid tumors [1], with 50–70% of cancer patients receiving RT during their care. However, 20–50% of these patients develop radioresistance, which is a major cause of therapeutic failure [2, 3]. While RT‐generated reactive oxygen species (ROS) induce DNA strand breaks and lipid peroxidation in oxygenated tumor cells, leading to direct cell death, residual hypoxic tumor cells often persist post‐treatment [4, 5]. Within these hypoxic niches, radiation stimulates the overexpression of pro‐survival factors (e.g., HSP70, GRP78, HIF‐1α), fostering radioresistance [6, 7, 8]. Compounding this issue, robust DNA repair mechanisms enable tumor cells to recover from radiation‐induced damage [9, 10]. Thus, strategies to alleviate tumor hypoxia and disrupt DNA repair are critical for overcoming radioresistance [11].

Nitric oxide (NO) is a potent gaseous signaling molecule, exhibiting well‐characterized vasodilatory effects leveraged in cardiovascular therapeutics (e.g., via nitroglycerin or isosorbide dinitrate). Recent studies reveal that NO synergistically enhances RT efficacy [11, 12]. Wang et al. found that NO vasodilation alleviates hypoxia, further sensitising cancer cells to radiotherapy, which plays an important role in mitigating tumor hypoxia and enhancing radiosensitivity [13]. Specifically, NO reacts with RT‐generated ROS to form peroxynitrite (ONOO^−^), which amplifies tumor DNA damage and suppresses DNA repair pathways [14, 15]. Pioneering work demonstrates ONOO^−^’s antitumor potential [16, 17]. Nevertheless, systemic NO delivery lacks tumor selectivity, causing off‐target toxicity and limiting clinical utility [18, 19, 20]. Xie et al. confirmed that NO could co‐suppress PDL1 and COX‐2 expression, revealing a potentially underrecognized role of NO in reversing tumor immunotherapy resistance [21]. Huang et al. constructed a platform named NBS‐2S‐NO designed to induce ferroptosis in cancer cells via ONOO^−^ generation, thereby overcoming limitations of traditional therapies imposed by the solid tumor microenvironment, such as hypoxia and elevated glutathione concentrations [22]. Ye et al. developed a supramolecular engineering‐based nanoplatform CSC_CPT/SNAP_ that enhances the tumor oxidative stress microenvironment through self‐supplying reactive species (ROS+NO), inducing pyroptosis and activating long‐term antitumor immunity to address the short lifespan and limited diffusion range of conventional ROS therapies [23, 24]. Yang et al. engineered a melanoma‐specific peroxynitrite nanoparticle APAP‐P‐NO, which reverses immunosuppressive tumor microenvironments by disrupting cancer cell metabolic homeostasis, thus potentiating immune checkpoint blockade therapy for melanoma treatment [25].

**SCHEME 1:**
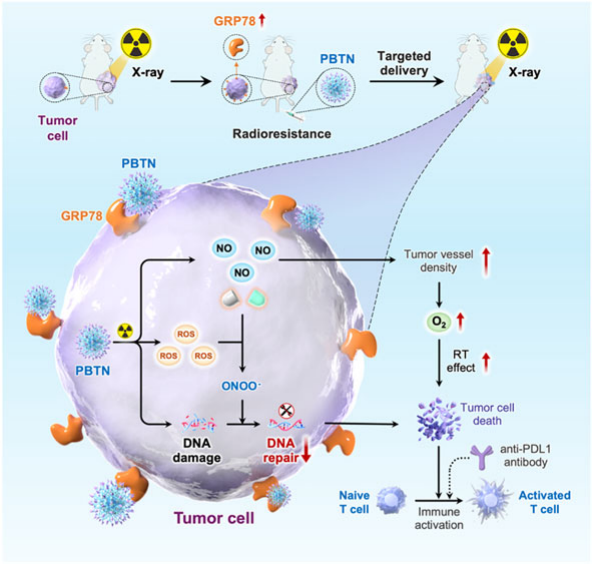
Schematic illustration of the antitumor mechanism of the radiation‐sensitive PBTN nanoplatform. RT induces the overexpression of GRP78 on the tumor cell surface. PBTN actively targets these cells, leading to increased intracellular generation of ONOO^−^ upon X‐ray irradiation. This enhances DNA damage while inhibiting repair pathways, ultimately killing tumor cells. The induced immunogenic cell death promotes the activation and tumor infiltration of CD8^+^ T cells, which in turn attack tumor cells. This combinatorial therapy amplifies tumor cell death and establishes a systemic antitumor immune response.

Here, we address this challenge through tumor‐targeted NO delivery designed to simultaneously alleviate hypoxia and block DNA repair (Scheme 1). We first identified BNN6 as an RT‐responsive NO donor, releasing NO upon radiation exposure. By encapsulating BNN6 within GRP78‐targeted nanoparticles (exploiting the overexpression of GRP78 in radioresistant tumors). We have successfully developed PEG‐PCL‐GRP78‐targeted nanoparticles (PBTN) composed of PEG‐PCL‐BNN6 (PBN), achieving active tumor‐specific delivery. Following localized irradiation (IR), BNN6 releases NO selectively within tumors. This spatiotemporally controlled release drives dual mechanisms: (1) NO‐mediated vasodilation ameliorates tumor hypoxia, and (2) NO‐derived ONOO^−^ cooperates with RT‐generated ROS to enhance DNA damage while inhibiting repair [26]. Combining active targeting with radiation‐triggered NO release establishes a precise and safe strategy for overcoming radioresistance [27].

## 2 Results

### 2.1 Design, Synthesis, and Characterization of Radioresistant‐Tumor‐Targeted Nanoparticle

**FIGURE 1:**
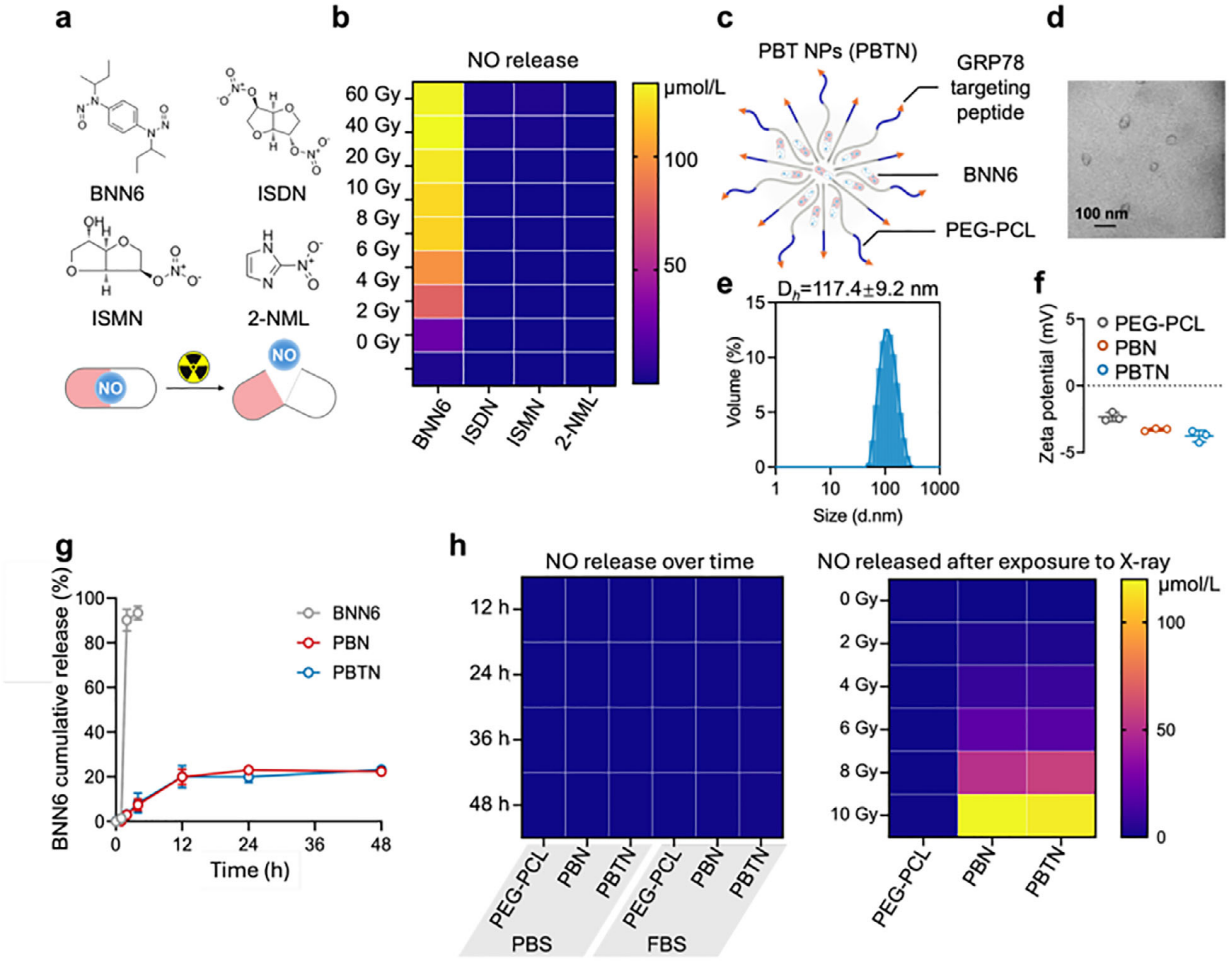
(a) The chemical structural formulae of BNN6, ISDN, ISMN, and 2‐NML. (b) Heat maps of NO release from BNN6, ISDN, ISMN, and 2‐NML under 0–60 Gy radiation at 25°C. (c) Schematic diagram of PBT NPs (PBTN). (d) TEM image of PBTN, Scale bar = 100 µm. (e) DLS results of PBTN. (f) Zeta potential of PEG‐PCL, PBN, and PBTN (*n* = 3). (g) BNN6 cumulative release of PBN and PBTN (*n* = 3) at 25°C. (h) NO release at different time points in different environments by PEG‐PCL, PBN, and PBTN in phosphate‐buffered saline (PBS) and fetal bovine serum (FBS) (left). NO release at different RT doses after treatment of PEG‐PCL, PBN, and PBTN (right). The reaction was carried out at 37°C.

To identify the most suitable radiosensitive NO donor small molecules, we measured NO release from N,N′‐di‐sec‐butyl‐N,N′‐dinitroso‐1,4‐phenylenediamine (BNN6), Isosorbide dinitrate (ISDN), Isosorbide Mononitrate (ISMN), and 2‐Nitroimidazole (2‐NML) after different radiation doses were administered (Figure 1a). At 0 Gy, all four compounds released minimal NO (Figure 1b). BNN6 demonstrated controlled NO release kinetics, with the cumulative NO concentrations increasing proportionally to the radiation dose until they reached a saturation threshold at 8 Gy, which indicated complete activation of the radiation‐sensitive functional groups. Although ISDN, ISMN, and 2‐NML showed a similar dose‐dependent trend, their NO release levels remained negligible and therapeutically insufficient. Based on these findings, we selected BNN6 as the optimal NO donor. We subsequently synthesized PEG‐PCL‐BNN6 nanoparticles and modified their surface with a GRP78‐targeting peptide to obtain PBT nanoparticles (PBTN) (Figure 1c). Dynamic light scattering (DLS) experiments revealed average hydrodynamic diameters of 28.3 ± 4.9 nm (PEG‐PCL), 116.5 ± 8.1 nm (PBN), and 117.4 ± 9.2 nm (PBTN) (Figure 1e; Figure S1). Transmission electron microscopy (TEM) imaging (Figure 1d) confirmed the particle size of PBTN of 58.4 ± 23.1 nm, which was smaller than the DLS‐measured hydrodynamic diameter owing to the absence of hydration effects. The zeta potentials were −2.34 ± 0.38 mV (PEG‐PCL), −3.28 ± 0.12 mV (PBN), and −3.77 ± 0.48 mV (PBTN) (Figure 1f). These properties ensured the suitability of the nanoparticles for in vivo application as an injectable depot.

To evaluate the stability and radiation‐triggered release profiles of PBN and PBTN nanoparticles, we quantified the retention of BNN6 (the NO donor) and monitored NO release under physiological and irradiated conditions. In PBS (pH 7.4), both PBN and PBTN exhibited excellent retention of encapsulated BNN6, with cumulative release rates of only 22.4 ± 1.8% (PBN) and 23.2 ± 2.1% (PBTN) over 48 h (Figure 1g). Concurrent experiments for measuring NO release in PBS and FBS solutions revealed minimal baseline leakage (<5 µmol/L) between 12 and 48 h (Figure 1h), confirming the stability of the nanoparticles in the absence of external stimuli. In contrast, following irradiation (IR, 8 Gy), a marked increase in NO release was observed. Within 12 h of irradiation, the NO concentration surged to 45.3 ± 3.7 µmol/L (PBN) and 49.6 ± 4.2 µmol/L (PBTN). It reached saturation levels of 70.1 ± 5.3 µmol/L (PBN) and 73.8 ± 6.1 µmol/L (PBTN) by 48 h. Figure S2 shows that NO release amounts of PBTN at different concentrations (0.05–5.0 mmol/L) after radiation therapy. This radiation‐dependent activation aligns with the designed mechanism of BNN6 decomposition, wherein radiotherapy‐generated ROS cleave the nitroimidazole‐derived NO donor bonds. Collectively, these results demonstrate that PBN and PBTN maintain structural integrity under physiological conditions while enabling precise, irradiation‐activated NO release, which is a critical feature for minimizing off‐target toxicity and enhancing therapeutic specificity.

### 2.2 X‐Ray Irradiation (IR) Controlled Release of NO From BNN6, PBN, and PBTN

**FIGURE 2:**
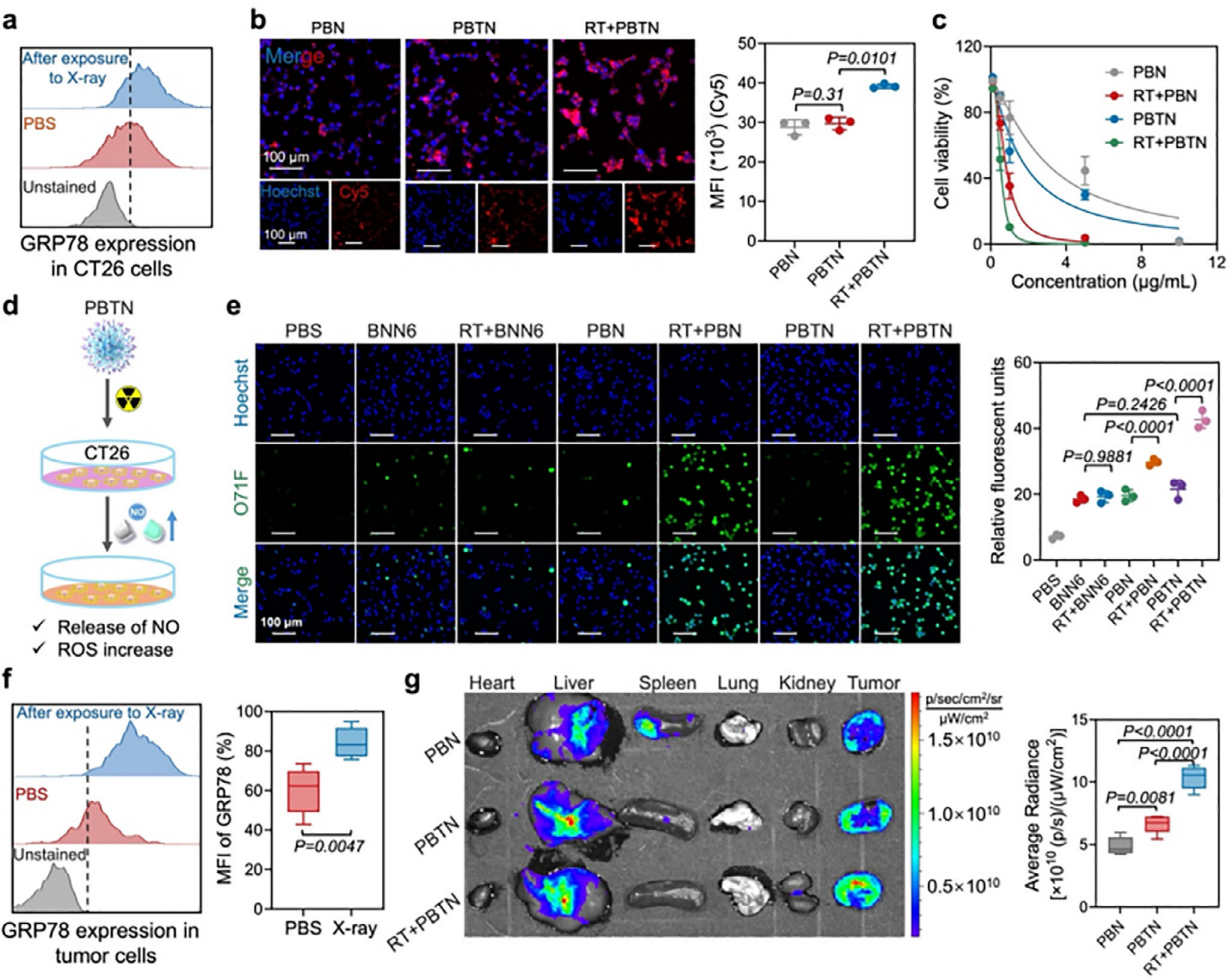
(a) GRP78 expression in CT26 cells subjected to RT. (b) The targeting potential of CT26 tumor cells and the fluorescence quantification of Cy5 in the PBN, PBTN, and RT+PBTN groups (at 4 h after 2 Gy irradiation). Hoechst‐labeled CT26 (blue) and Cy5‐labeled PBN/PBTN (red) cells. (c) Relative viability of CT26 cells treated with PBN, IR+PBN, PBTN, and IR+PBTN at different concentrations for 24 h (*n* = 3). (d) Schematic representation of the experiment for detecting NO and ROS release. (e) In vitro fluorescence images depict NO release and fluorescence quantification in the PBS, BNN6, IR+BNN6, PBN, IR+PBN, PBTN, and IR+PBTN groups. Hoechst‐labeled CT26 cells (blue). O71F‐marked NO (green). (f) GRP78 expression in CT26 tumor tissue cells; MFI of GRP78 in PBS and RT. (g) The distribution and quantitative analysis of Cy5‐labeled PBN/PBTN in vivo experiments (*n* = 3).

GRP78 expression in the CT26 and HT29 cells was upregulated 4 h after IR of X‐ray (0.5 Gy, Figure 2a; Figures S3 and S4). First, the ability of PBTN to control NO release in vitro was evaluated. To verify whether the synthesized PBN and PBTN could be internalized by CT26 cells, we co‐incubated the PBN/PBTN with CT26 cells for 4 h. As shown in Figure 2b, for tumor cells not exposed to pre‐IR, PBN and PBTN show insignificant tumor targeting. However, in tumor cells irradiated with pre‐IR, PBTN achieves significantly enhanced tumor targeting due to the increased expression of GRP78. Therefore, the GRP78‐targeting peptide modified on PBTN is more effective in delivering the drug to the tumor site after irradiation. To verify the sensitivity of CT26 cells to NO, the cytotoxic effect of PBN and PBTN on CT26 cells under radiotherapeutic conditions was detected using the CCK‐8 assay. All NO‐containing materials exert varying degrees of cytotoxic effects on CT26 cells (Figure 2c). CT26 cells were co‐incubated with PBN or PBTN for 24 h after radiotherapeutic treatment. Both PBN and PBTN groups showed increased toxicity with increasing dosage. In the PBTN group, the difference in the IC_50_ values before and after irradiation was statistically significant (0.17 µg/mL (pre‐RT) and 0.06 µg/mL (post‐RT), respectively). Irradiation of CT26 cells that were treated with PBTN led to the release of NO, thus enhancing the killing ability of tumor cells. The toxicity of the IR+PBN (IC_50_ = 0.14 µg/mL) and IR+PBTN groups was significantly higher than that of the PBN (IC_50_ = 0.57 µg/mL) and PBTN groups. These results prove that IR can significantly increase the toxic effects of PBN or PBTN on tumor cells. The IR+PBTN group achieved a strong cellular killing effect.

The green fluorescence results of NO indicated that the IR+PBTN group released the highest levels of NO, which was 1.43 times that observed in the IR+PBN group (Figure 2d,e). Lower levels of NO were released in the BNN6, IR+BNN6, PBN, and PBTN groups. This confirmed that NO release from PBTN increased significantly after irradiation.

Analysis of the cellular GRP78 expression results obtained using flow cytometry (Figure 2f) revealed a significant increase in GRP78 expression in the irradiated group, indicating a cellular stress response to radiation exposure. Based on these findings, we proceeded with the subsequent experiments. In vivo fluorescence imaging revealed significant fluorescence present exclusively in the tumor of mice treated with Cy5‐labeled PBTN after treatment, in contrast to minimal or undetectable fluorescence in the other organs (Figure 2g). The PBN and PBTN groups generated lower levels of NO, but a large volume of NO was synthesized in response to radiotherapy. The RT+PBTN group showed a markedly stronger fluorescence intensity in the tumor region, which was 1.56 times that in the PBTN group. This confirmed that radiotherapy can selectively induce NO release from PBTN and cause more severe DNA damage. The presence of NO was shown to inhibit DNA repair in cells.

### 2.3 Evaluation of In Vivo Antitumor Effect

**FIGURE 3:**
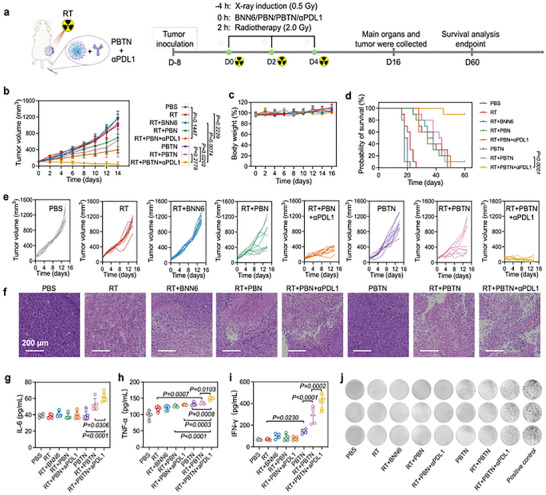
PBTN enhanced the abscopal effect of radiotherapy. (a) Schematic representation of the tumor treatment protocol used in mice. (b) Tumor volume curves in mice that underwent different treatments (PBS, RT, RT+BNN6, RT+PBN, RT+PBN+αPDL1, PBTN, RT+PBTN, RT+PBTN+αPDL1) (*n* = 10 per group). The drug injection was performed 4 h post‐IR (0.5 Gy). (c) Body weight of mice subjected to different treatments. (d) Survival curves of mice subjected to different treatments. (e) Tumor volume curves of each group after treatment in 15 days. (f) H&E staining of tumor sections subjected to different treatments (PBS, RT, PBTN, RT+PBTN, and RT+PBTN+αPDL1). (g) IL‐6, (h) TNF‐α, and (i) IFN‐γ levels in tumor tissue of different treatments were measured by ELISA (*n* = 5). (j) IFN‐γ ELISPOT analysis of blood samples under different treatments and a positive control (*n* = 3).

Owing to the excellent performance of PBTN in vitro, its antitumor effect was investigated in BALB/c mice in vivo. A tumor‐bearing mice model was established via the subcutaneous injection of CT26 cells (Figure 3a). Tumor cells were injected into the right flank of mice. When the tumor volume was approximately 100 mm^3^, the mice were randomly divided into eight groups (PBS, RT, RT+BNN6, RT+PBN, RT+PBN+αPDL1, PBTN, RT+PBTN, and RT+PBTN+αPDL1), and received 0.5 Gy X‐ray 4 h before drug injection to induce radioresistance. Then, 2 h after the drug was injected, mice received RT (2.0 Gy). The tumor volume and body weight were recorded every other day and after all treatments were administered. Tumor growth was most potently suppressed in the RT+PBTN+αPDL1 group, resulting in a tumor inhibition rate of 96.5% (*p* < 0.0001 vs. PBS, Figure 3b,e). Concurrently, a parallel long‐term survival study was conducted where mice were monitored for 60 days without sacrifice (mice were sacrificed when tumor volume exceeded 1500 mm^3^). The survival of mice was prolonged significantly. Compared to that in the PBS group, more than 80% of the mice in the αPDL1 group survived for an additional 40 days (Figure 3d).

However, the images of stained tumor sections showed obvious damage to tumor tissues in the PBTN group (Figure 3f), which confirmed an effective tumor suppression effect exerted by this tumor‐specific NO platform with favorable biosafety.

### 2.4 RT+PBTN+αPDL1‐Derived Immunosuppressive Tumor Microenvironment Reversion Effect in CT26 Tumors

Subsequently, Elisa and ELISPOT assays were conducted on mouse spleen tissues. As shown in Figure 3g–i, the RT+PBTN+αPDL1 group had the highest levels of IL‐6, TNF‐α, and IFN‐γ. After RT+PBTN+αPDL1 treatment, the concentration of IL‐6 and TNF‐α increased by 1.6 and 1.5 times, respectively. The serum levels of these cytokines were positively correlated with their levels in tumor tissues. The IFN‐γ level was significantly higher in RT+PBTN+αPDL1‐treated mice than in PBS‐treated ones, further indicating immune activation. ELISPOT of tumor tissues also revealed a significant increase in the level of IFN‐γ in the RT+PBTN+αPDL1 group (Figure 3j).

**FIGURE 4:**
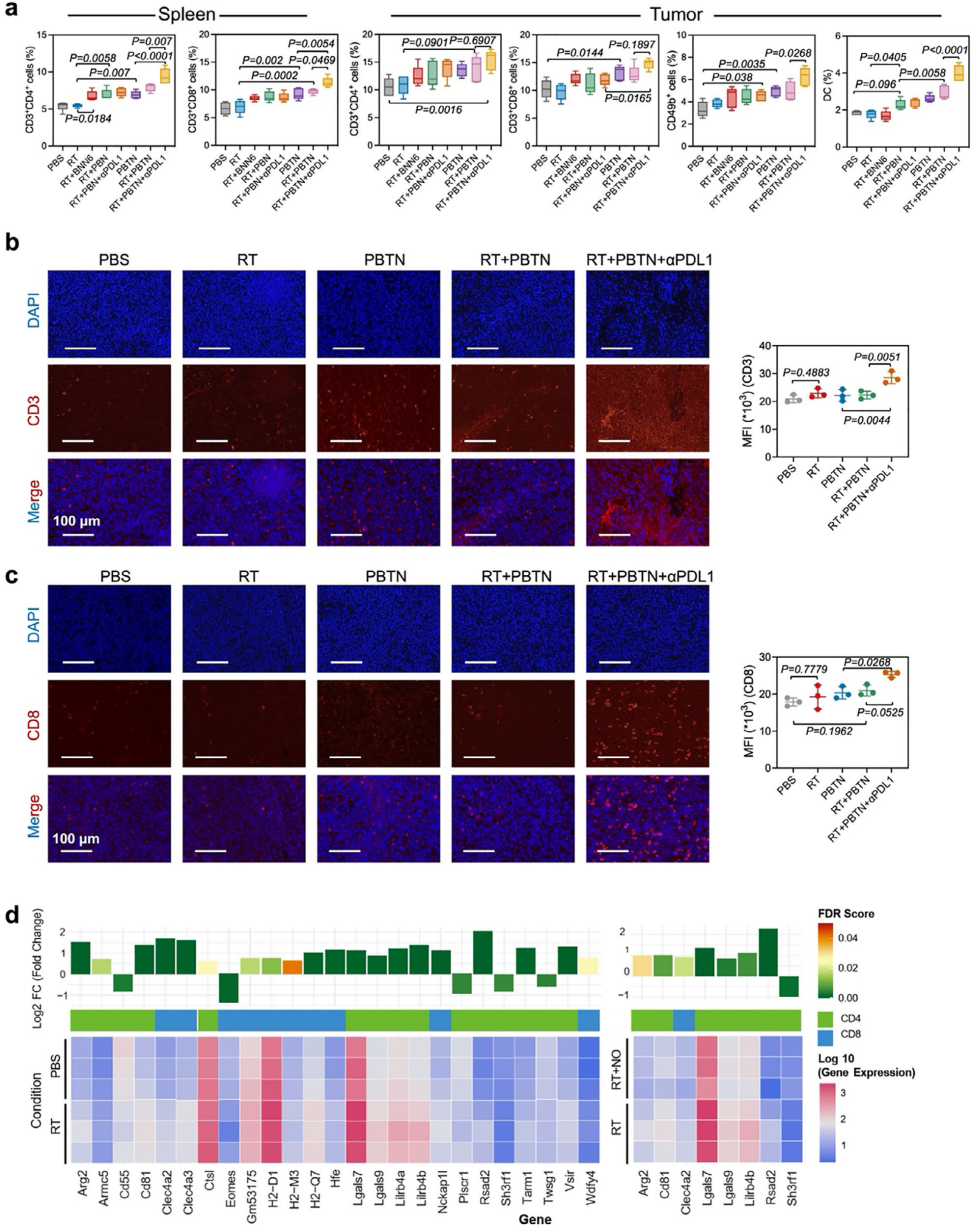
(a) The accumulation of CD4^+^/CD8^+^ T cells in the spleen of mice induced in response to the treatments was measured using flow cytometry (left). The accumulation of CD4^+^ T cells, CD8^+^ T cells, CD49b^+^ cells, and DC cells in mice tumors induced in response to the treatments were measured using flow cytometry. (b) Immunofluorescence staining showed the presence of CD3^+^ and CD8^+^ T cells in the tumors of mice subjected to different treatments (PBS, RT, PBTN, RT+PBTN, and RT+PBTN+αPDL1). (c) The expression intensities of CD4 and CD8 in mouse tumors after treatment with PBS, RT, and RT+PBTN+αPDL1, respectively (*n* = 3). Scale bar = 100 µm. (d) Heatmap and bar plot show the expression and fold change of selected immune‐related genes across groups (RT&PBS, RT&RT+NO). Colors represent scaled expression (bottom) and log2 fold change (top) values. The BNN6, PBN, and PBTN injections were performed 4 h post‐IR (0.5 Gy).

To further elucidate the mechanism underlying the superior tumor inhibition effect in the RT+PBTN+αPDL1 group, tumor immunophenotypic profiles were analyzed using flow cytometry [28, 29, 30, 31]. Compared to that in the PBTN group, the spleen and tumor tissues of mice in this group had a greater abundance of CD3 and CD8 cells (Figure 4a; Figures S7–S9). The abundance of dendritic cells (DCs) and natural killer cells (NK) in the tumor tissues was also higher (Figure 4a; Figures S10 and S11). The abundance of CD8^+^ T cells increased from 6.6% to 11.3% (*p <* 0.0001 vs. PBS) compared with that in the PBS treatment group. Flow cytometry analysis of tumor tissue cells revealed a significant increase in the abundance of CD4^+^ T cells, CD8^+^ T cells, CD49b^+^, and DC cells in the RT+PBTN+αPDL1 group. Immunofluorescence analysis of tumor sections revealed markedly enhanced CD3^+^/CD8^+^ signals in the RT+PBTN+αPDL1 group compared to other treatment cohorts (Figure 4b,c). The same result is presented in the genomic heat map shown in Figure 4d.

**FIGURE 5:**
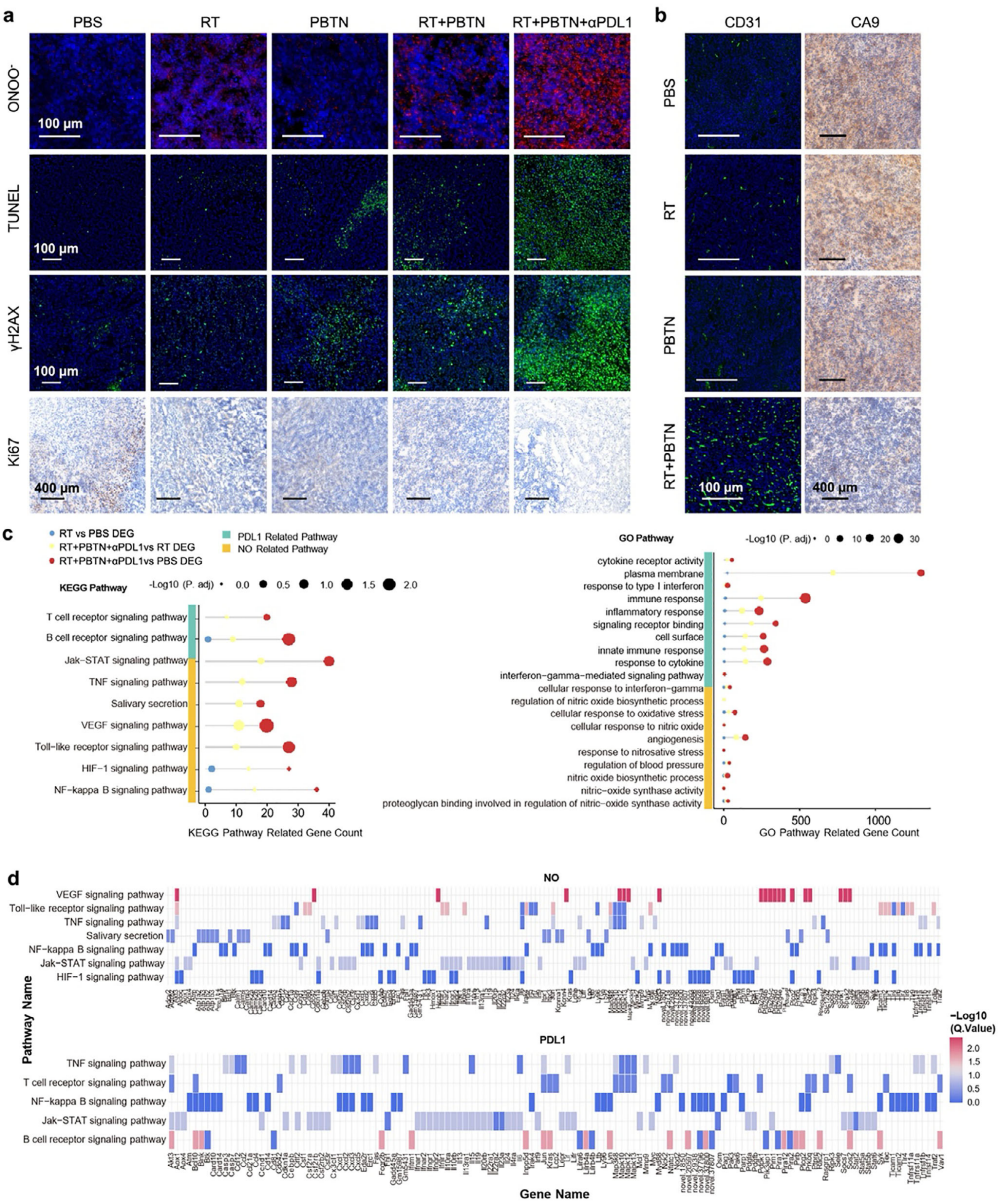
(a) ONOO^−^, TUNEL, γH2AX, and Ki67 staining of tumor sections subjected to different treatments (PBS, RT, PBTN, RT+PBTN, and RT+PBTN+αPDL1). (b) CD31 and CA9 staining of tumor sections after treatment with PBS, RT, PBTN, and RT+PBTN. (c) KEGG and GO‐related gene count of PBS, RT, and RT+PBTN+αPDL1 (*n* = 3). (d) The significance of KEGG pathway‐related genes in the NO pathway and PDL1 in tumors treated with PBS, RT, and RT+PBTN+αPDL1 (*n* = 3). The PBTN injection was performed 4 h post‐IR (0.5 Gy).

Given the established role of tumor cell metabolism in shaping the immunosuppressive tumor microenvironment and the profound metabolic impact of NO, we investigated how RT+PBTN+αPDL1 treatment remodeled the immune landscape in CT26 tumors. Based on the robust metabolic interference exhibited by NO, we hypothesized that the immune phenotype of CT26 tumor cells and the tumor microenvironment may be substantially affected upon treatment with this ONOO^−^‐generating material [32, 33]. To verify this, we first evaluated the differentially expressed genes related to immune regulation functions in CT26 tumor cells by analyzing the transcriptome data (Figures 4d and 5c,d). The flow cytometry analysis of tumor and spleen cells yielded similar results.

### 2.5 Tumor Tissue Analysis and Immune Mechanism Investigation

ONOO^−^ staining in tissue sections showed that the PBTN group had the highest ONOO^−^ content [33]. To assess the impact of ONOO^−^ on immune function, immunocyte staining was performed on tumor tissues (Figure 5a; Figure S5). TUNEL staining, which helps detect apoptosis, showed the highest signal intensity in the RT+PBTN+αPDL1 group, suggesting the most extensive cell death in this group (Figure 5a). Immunofluorescence staining for γH2AX (a marker of DNA double‐strand breaks) showed the most intense and widespread foci in the RT+PBTN+αPDL1 group, indicating severe DNA damage and impaired cell repair capacity in this group (Figure 5a). Consistent with this, Ki67 staining exhibited the lowest proliferative index in this group [34], further corroborating the potent antitumor effect (Figure 5a). Staining for CA9 (a hypoxia marker) was significantly weaker in the RT+PBTN groups compared to that in the controls, indicating effective hypoxia alleviation (Figure 5b). Hypoxia is a known microenvironment factor that promotes metastatic progression [35]. CD31 staining revealed enhanced vascular density in the RT+PBTN groups (Figure 5b; Figure S6), potentially indicating improved vascular normalization in response to NO release.

Compared with that in the RT group, the expression of NO‐ and PDL1‐related genes in the PBTN+PDL1 group increased significantly (Figure 5c,d). Pathway analysis showed that radiotherapy‐associated DNA damage primarily activated NF‐κB, TNF, Jak–STAT, and HIF‐1 signaling in the NO condition, consistent with a DNA damage‐induced inflammatory stress response (Figure 5d). In contrast, the PDL1 condition was characterized by enrichment of T cell receptor signaling, together with NF‐κB, Jak–STAT, and TNF pathways, indicating enhanced T cell‐mediated immune activation. Notably, NF‐κB and Jak‐STAT signaling represented shared hubs linking DNA damage responses to adaptive immunity, supporting a functional coupling between radiotherapy‐induced genotoxic stress and T cell engagement. Collectively, these data provide evidence that RT+PBTN+αPDL1 can activate antitumor adaptive immunity. Simultaneously, these findings prove that NO and PDL1 exert synergistic radiosensitizing effects when exposed to X‐ray irradiation [36, 37].

### 2.6 Biocompatibility and Pharmacokinetics of PBTN

The favorable in vitro and in vivo biocompatibility of PBTN guaranteed the smooth biological evaluation of these materials. We also assessed the potential toxicity of PBTN in vivo to validate its clinical and translational potential. Hematological data from mice intravenously injected with PBTN were collected and analyzed. As shown in Figure S12, liver function indicators, including aspartate transaminase (AST), alanine transaminase (ALT), alkaline phosphatase (AKP), blood urea nitrogen (BUN), creatinine (CRE), and urea (UA), were comparable in mice from the PBTN and PBS groups. Moreover, none of the experimental groups showed a significant reduction in body weight compared to the PBS group (Figure 3c). In the RT+PBTN+αPDL1 group, the examination of major organs, including the liver, spleen, kidneys, heart, and lungs, revealed no apparent damage or inflammatory lesions (Figure S13). These results indicate that PBTN exhibits favorable biocompatibility and satisfactory in vivo therapeutic effects. Therefore, the novel nano‐radiosensitizer PBTN containing NO and GRP78‐targeting peptide may have considerable potential for future clinical translation to achieve effective radio‐immunotherapy.

## 3 Discussion

Radiotherapy resistance, fueled by tumor hypoxia and enhanced DNA repair mechanisms, remains a significant barrier to effective cancer treatment [38, 39]. Current strategies to overcome this resistance, including the use of NO to ameliorate hypoxia and generate DNA‐damaging peroxynitrite (ONOO^−^), are hampered by systemic toxicity due to the lack of tumor specificity in NO delivery [37]. Our study directly addresses this critical limitation by developing PBTN, a GRP78‐targeted nanoplatform designed for selective tumor accumulation and X‐ray‐triggered NO release.

This approach strategically exploits the radiation‐induced overexpression of GRP78 in resistant tumors to achieve active targeting, establishing PBTN as a precise therapeutic platform for enhanced radiotherapy. The encapsulation of the radiosensitive NO donor BNN6 within GRP78‐targeted nanoparticles ensures minimal off‐target release, as evidenced by the significantly higher intertumoral fluorescence intensity (1.56‐fold) observed with PBTN compared to nontargeted controls. Crucially, irradiation within the tumor volume specifically triggers BNN6 decomposition, generating NO locally. This spatiotemporally controlled NO release executes a dual mechanism: it alleviates hypoxia through vasodilation and reacts with radiotherapy‐generated ROS to form cytotoxic ONOO^−^ [37, 40]. Our data confirm that ONOO^−^ not only amplifies DNA damage (as shown by intense γH2AX foci) but also potently suppresses DNA repair pathways, thereby functioning as a potent radiosensitizer. This synergistic action resulted in significantly enhanced tumor cell killing, as demonstrated by the lower IC_50_ post‐RT in vitro, which translated into dramatic tumor growth suppression (96.5% inhibition) and extended survival (>80% at 40 days) in vivo with the RT+PBTN+αPDL1.

The potent antitumor effects extend beyond direct cytotoxicity. Our results reveal that the RT+PBTN+αPDL1 combination profoundly reshapes the immunosuppressive tumor microenvironment. This is evidenced by the robust increase in pro‐inflammatory cytokines (IL‐6, TNF‐α, IFN‐γ), elevated infiltration and activation of cytotoxic CD8^+^ T cells, NK cells, and DCs within the tumor, and enhanced splenic T cell populations [41, 42, 43, 44]. This immune activation, coupled with the reversal of hypoxia and suppression of DNA repair, creates a synergistic platform that sensitizes tumors to both radiotherapy and checkpoint blockade, collectively sensitizing tumors to radio‐immunotherapy [45, 46]. The favorable biocompatibility profile of PBTN, demonstrated by the absence of significant weight loss, normal hematological parameters, and lack of major organ damage, highlights its potential for clinical translation. Consequently, the GRP78‐targeted, radiation‐triggered NO nanogenerator PBTN represents a safe, precise, and highly translatable strategy to overcome radioresistance and potentiate the efficacy of combined radio‐immunotherapy. Future studies integrating multi‐omics analyses will further elucidate the metabolic and immune reprogramming induced by tumor‐specific NO stress.

In conclusion, our GRP78‐targeted, radiation‐triggered NO nanogenerator PBTN represents a safe, precise, and highly translatable strategy that concurrently addresses the key pillars of radioresistance: hypoxia, DNA repair, and immunosuppression. Future studies will employ multi‐omics analyses to delineate the metabolic and immune reprogramming induced by tumor‐specific NO/ONOO^−^ stress and will validate this platform in more complex, clinically relevant models, such as spontaneous metastatic or patient‐derived xenograft models.

## 4 Experimental Section

Monomethoxy polyethylene glycol (mPEG_5k_, average molecular weight = 5.0×10^3^ g∙mol^−1^) was supplied by Sigma–Aldrich (Beijing, China). It was recrystallized from ethyl acetate and dried under vacuum before use. 1,1′‐(Hexane‐1,6‐diyl) bis (5‐nitropyrimidine‐2,4,6(1H,3H,5H)‐trione) (BNN6), isosorbide mononitrate (ISMN), isosorbide dinitrate (ISDN), and 2‐nitroimidazole (2‐NMI) were purchased from Sigma–Aldrich. (4,5‐Dimethylthiazol‐2‐yl)‐2,5‐diphenyltetrazolium bromide (MTT) was obtained from GL Biochem (Shanghai, China). GRP78‐targeting peptide (GRP78: WIFPWIQLC) was obtained from Seebio Biotech Co., Ltd. (Shanghai, China). DAF‐FM Diacetate (DAF‐FM DA) was purchased from Molecular Probes (Shanghai, China). Roswell Park Memorial Institute (RPMI) 1640 culture medium and fetal bovine serum (FBS) were acquired from Gibco (New York, USA). An anti‐CD31 antibody was purchased from Santa Cruz Biotechnology (Texas, USA). FITC‐anti‐CD3, anti‐CD4, and anti‐CD8 antibodies were obtained from BD Bioscience Pharmingen (San Diego, CA, USA). Hoechst 33342, anti‐γH2AX, and a ROS assay kit were purchased from Beyotime (Shanghai, China). A peroxynitrite (ONOO^−^) fluorescent probe was obtained from BestBio (Shanghai, China). ELISA kits for AST, ALT, AKP, BUN, CRE, and UA were purchased from Nanjing Jiangcheng Bioengineering Institute Co., Ltd. (Nanjing, China).

### 4.1 Synthesis and Preparation of PEG‐PCL, PBN, and PBTN

A mixture of 1 g (0.2 mmol) of PEG, 2 g (17.5 mmol) of ε‐caprolactone, and 5 mg of stannous octoate (Sn(Oct)_2_) was dissolved in anhydrous toluene in a dried flask. The solution was heated under reflux for 2 h to remove trace water by azeotropic distillation. The solvent was then removed under reduced pressure. The polymerization was performed at 120°C for 24 h under a nitrogen atmosphere. The resulting product was dissolved in dichloromethane (CH_2_Cl_2_) and precipitated into an excess of cold diethyl ether. The precipitate was collected by filtration and dried under vacuum to yield 1.8 g of PEG‐PCL‐OH as a white solid. 1 g (approximately 0.1 mmol) of PEG‐PCL‐OH was dissolved in 10 mL of anhydrous tetrahydrofuran (THF). To this solution, 28.6 mg (0.15 mmol) of p‐toluenesulfonyl chloride (TsCl) was added slowly at 0°C. The reaction was stirred at 0°C for 6 h. Subsequently, a large excess of ethylenediamine was added, and the temperature was raised to 50°C to react overnight. After the reaction, the mixture was poured into a large volume of cold diethyl ether to precipitate the product. The precipitate was collected by centrifugation and washed three times by re‐dissolving in a minimal amount of THF and reprecipitating in cold ether. The final product, PEG‐PCL‐NH_2_, was obtained after drying under vacuum. 1 g (approximately 0.1 mmol) of PEG‐PCL‐NH_2_ was dissolved in 5 mL of anhydrous N,N‐Dimethylformamide (DMF). In a separate vial, N‐(ε‐Maleimidocaproic acid) succinimide ester (EMCS, 1.2 equivalents) was dissolved in a small volume of anhydrous DMF. This solution was added dropwise to the stirred polymer solution under a nitrogen atmosphere and in the dark. Then, 20 µL of N,N‐Diisopropylethylamine (DIPEA) was added as an acid scavenger. The reaction was allowed to proceed at room temperature for 24 h with continuous stirring. The reaction mixture was transferred into a dialysis bag (MWCO: 3500 Da) and dialyzed against deionized water for 48 h, with water changed every 6 h. The final product, Mal‐PEG‐PCL, was obtained as a white solid after freeze‐drying.

Mal‐PEG‐PCL (10 mg) and BNN6 (1 mg) were co‐dissolved in 1 mL of acetone. This organic solution was injected into 20 mL of vigorously stirred deionized water using a syringe. The mixture was stirred for 6 h at room temperature to allow the organic solvent to evaporate spontaneously. The crude nanoparticle suspension was then purified by ultrafiltration centrifugation at 4°C and 10 000 × g for 15 min. This washing process was repeated three times with deionized water to remove unencapsulated BNN6. The resulting nanoparticles, designated PBN, were collected and stored at 4°C for immediate use.

Ten milligrams of PBN nanoparticles were dispersed in 5 mL of PBS (pH 7.4). Then, 2.4 mg of GRP78‐targeting peptide was added. The conjugation reaction was carried out at 4°C for 6 h under gentle stirring. Unreacted peptides were removed by ultrafiltration centrifugation (100 kDa MWCO, 4°C, 10 000 × g, 10 min) for three cycles. The final product, the GRP78‐targeted nanoparticle PBTN, was obtained and stored at 4°C.

### 4.2 Characterization

The nanoparticle size and zeta potential were measured using DLS, which was performed on a Malvern Zetasizer instrument (Nano‐ZS90). The immunofluorescence slides and cellular uptake slides were viewed through a confocal laser scanning microscope (CLSM, Carl Zeiss LSM 700). Transmission electron microscope (TEM) imaging was performed on a JEOL JEM‐1011 transmission electron microscope (Tokyo, Japan) with an accelerating voltage of 100 kV.

### 4.3 Cells and Animals

The murine colon cancer cell line CT26 and the fibroblast NCTC clone 929 cell line (L929) were procured from Shanghai Bogoo (Shanghai, China). The CT26 and L929 cells were cultured in RPMI 1640 medium with a high glucose level and supplemented with 10% FBS, 1% penicillin, and 1% streptomycin and incubated at 37°C in an atmosphere with 5% CO_2_ and 95% air (approximately 20% O_2_ equivalent). The human colon cancer cell line HT29 was procured from Procell (Wuhan, China) and cultured in CM‐0118 specialized medium under the same environment.

CT26 tumor cells were inoculated in 6‐well plates at a density of 7 × 10^4^ cells per well. After 6 h of incubation, a fresh medium containing BNN6, PBN, or PBTN (10.0 mg/mL) was added to each well. Subsequently, radio‐stimulation was performed at doses of 2, 4, 6, 8, 10, 20, 40, and 60 Gy (1 Gy/min). This was followed by centrifugation. Lastly, 50 µL each of Griess Reagent I and Griess Reagent II was added to the cells. The NO content was measured at 540 nm.

Irradiation was performed using an RS2000 Pro‐225TR X‐ray irradiator (RadSource Technologies, GA, USA). For cell lines, cells in 6‐well plates or T25 flasks were irradiated at a source‐to‐surface distance (SSD) of 50 cm with a 20 cm × 20 cm field, delivering 1.0 Gy/min. For animal studies, tumors were centered in a 10 cm × 10 cm collimated field with lead shielding of nontarget tissues, using the same SSD and dose rate. Dose calibration was performed using an ionization chamber (PTW, Freiburg, Germany) connected to an electrometer, and verified with radiochromic film (Gafchromic EBT3, Ashland Inc., USA) or optically stimulated luminescence dosimeters (nanoDot, Landauer Inc., USA). Sham‐irradiated controls were placed in the irradiator for the same duration without X‐ray exposure.

All experiments involving animals and procedures of animal care and handling were performed in accordance with protocols approved by the guidelines of the Animal Ethics and Welfare Committee of Jilin University (Ethic Approval No. 2025700). Female BALB/c mice (6–8 weeks old) were purchased from Beijing Vital River Laboratory Animal Technology Co., Ltd. (Beijing, China). The mice were housed in an institutional pathogen‐free facility on a 12 h reverse light/dark cycle (7 PM–7 AM). The animal facility was maintained at a temperature of 23 ± 2°C with 55 ± 5% humidity. The mice were euthanized if their tumor volume exceeded 2000 mm^3^ or if they exhibited signs of distress, in accordance with institutional humane endpoint criteria.

### 4.4 ELISPOT Assay

CT26 tumor‐bearing mice were established using the process described in the section on CT26 tumor model establishment. Splenocytes were harvested for 5 days post‐treatment. Before the splenocytes were seeded into ELISPOT plates (BD Biosciences, Catalog Number 551083), the plates were pre‐wetted with RPMI‐1640 medium for 10 min and treated for 24 h with an IFN‐γ capture antibody at 4°C. Subsequently, the plates were blocked at room temperature for 2 h. Following this, 2 × 10^5^ splenocytes were seeded into each well and treated with antigens for 48 h at 37°C in a 5% CO_2_ and humidified incubator. Following this, the detection antibody was added to the wells after the plates had been washed with deionized water. The detection antibody was biotinylated anti‐mouse IFN‐γ used at 1:250 dilution. Spot visualization was performed by incubating the plate with streptavidin‐horseradish peroxidase HRP and then with an AEC/acetate substrate solution.

### 4.5 NO Intracellular Detection

DAF‐FM DA (Beyotime, S0019) was used to detect intracellular NO. Briefly, 2 × 10^5^ CT26 cells were seeded in each confocal dish and cultured at 37°C for 24 h. The cells were treated with DAF‐FM DA solution (1: 1000 dilution) for 30 min, washed with PBS, and then treated with 200 µmol/L PBTN at 37°C for 4 h. The cells were washed with cold PBS and imaged on a CLSM. Cells maintained in the blank culture medium were used as the control.

### 4.6 Intracellular ONOO^−^ Detection

An ONOO^−^ fluorescent probe (BestBio, BB‐460652) was used to detect intracellular ONOO^−^. Briefly, 2 × 10^5^ CT26 cells were seeded per well in confocal dishes and 6‐well plates and cultured at 37°C for 24 h. The cells were washed with PBS and then treated with 200 µmol/L BNN6, PBN, or PBTN at 37°C for 4 h after radiotherapy. Following this, the cells were stained with an ONOO^−^ fluorescent probe solution (1: 1000 dilution) for 30 min and washed with PBS. Finally, for qualitative analysis, the cells cultured in confocal dishes were washed with PBS and imaged on a CLSM. The cells in 6‐well plates were harvested, collected via centrifugation, suspended in PBS, and subjected to flow cytometry for quantitative analysis. Cells maintained in the blank culture medium were used as the control. The quantitative analysis test was performed three times.

### 4.7 Cytotoxicity Evaluation

The CCK‐8 assay was conducted to evaluate the cytotoxicity of the materials. For the CCK‐8 assay, 5 × 10^3^ CT26 cells were seeded per well in 96‐well plates and cultured at 37°C for 24 h. Following this, the culture medium was replaced with fresh medium containing BNN6, PBN, or PBTN at different concentrations (0, 10, 50, 100, 250, and 500 µmol/L). After the cells were incubated for 24 h, 10 µL of CCK‐8 solution was added to each well, and the mixture was incubated for another 2 h. Optical absorbance was measured at 450 nm using a Tecan Spark Microplate Reader (Switzerland).

### 4.8 Animal Experiments and Tumor Models

Colorectal tumor models were established by subcutaneously injecting CT26 cells (1 × 10^6^ in aseptic PBS) into the flank region of female BALB/c mice (4–6 weeks) for tumor treatment. Treatment commenced when the tumors showed an average volume of ∼100 mm^3^ (typically on day 6 post‐inoculation). Notably, tumor‐bearing mice were euthanized when the tumor volume exceeded 2000 mm^3^.

### 4.9 In Vivo Antitumor Study

To preliminarily evaluate the antitumor effect of various materials on colorectal tumors, CT26 subcutaneous tumor‐bearing BALB/c mice were randomly divided into eight groups (*n* = 10). Mice were injected intravenously with BNN6, PBN, or PBTN in PBS and pure PBS (with 5.5 mg/kg based on BNN6, 100 µL per mouse) on days 0, 2, 4, and 6. At 12 h after the last administration, one mouse in each group was euthanized to collect the tumor tissues for an intertumoral ONOO^−^ test using an ONOO^−^ assay kit (Bestbio, BB‐470569) as well as γH2AX detection through immunofluorescence analysis.

The tumor length (L), tumor width (W), and body weight of the mice were monitored on alternate days. The tumor size was calculated using this formula: volume = W^2^ × L / 2. On day 12, the remaining mice were sacrificed to obtain blood, tumor tissues, and major organs (heart, liver, spleen, lung, and kidney). Pathological analysis of the organs was conducted using hematoxylin and eosin (H&E) staining assays. The tumors were imaged and weighed for tumor inhibition rate calculations using this formula: tumor inhibition rate (%) = (mean tumor weight of PBS group‐tumor weight of treatment group)/(mean tumor weight of PBS group) × 100. The procedures and grouping of mice used in the survival analysis were consistent with those used in the preceding experiments. The health of each mouse was monitored daily during the study period. The mice were sacrificed when the tumor volume reached 2000 mm^3^.

### 4.10 H&E, TdT‐Mediated dUTP Nick‐End Labeling (TUNEL), and Immunofluorescence Staining

The mice were dissected. Their major organs (heart, liver, spleen, lung, and kidney) and tumor tissues were collected and fixed in 4% (W/V) PBS‐buffered paraformaldehyde and embedded in paraffin. The paraffin‐embedded tumor and organ tissues were cut into 5 µm thick sections for the H&E staining of the organs. H&E and TUNEL staining were performed using tumor tissues. Immunofluorescence staining for CD31, CA9, Ki67, CD3, and CD8 was performed using the paraffin‐embedded tumor tissues. Specifically, the tissue sections were placed in a repair box containing an EDTA (pH 8.0) antigen repair solution in a microwave oven for antigen repair. The block was treated with BSA for 30 min. The sections were treated with primary antibodies against CD and CD8 (added dropwise) and incubated overnight at 4°C. Following this, the sections were treated with Cy3‐labeled secondary antibodies corresponding to the primary antibodies and incubated in the dark at room temperature for 50 min. The nucleoli were stained with 4′,6‐diamidino‐2‐phenylindole (DAPI). Sections were observed under a fluorescence microscope, and images were recorded. Following this, the tumors were fixed in 4% formaldehyde for CD31 and CA9 detection using immunofluorescence analysis.

### 4.11 FACS Analysis

Changes in the immune microenvironment of mice subjected to different treatments were detected using flow cytometry. CT26 cells (6 × 10^5^) were injected into the abdomen of mice to establish a subcutaneous CT26 tumor model. When the tumor volume of the mice was approximately 200 mm^3^, the mice were randomly divided into eight groups and subjected to different treatments (*n* = 5). For CD8^+^ T, CD4^+^ T, DC, and NK cells detection, the cells were stained with PE‐anti‐CD45, FITC‐anti‐CD3, APC‐anti‐CD8, and APC/PE‐anti‐CD4 antibodies. Finally, all cells were analyzed using flow cytometry (BD, FACSCelesta). Blood samples were collected from the orbital on day 12. Sera were separated after centrifugation (1000 × g, 4°C for 20 min).

The levels of IL‐6, TNF‐α, and IFN‐γ in mouse tumor tissues were measured using LEGEND MAX ELISA Kits from BioLegend (IL‐6: Cat# 431304; TNF‐α: Cat# 430904; IFN‐γ: Cat# 430804), strictly in accordance with the manufacturer's protocols (*n* = 5).

### 4.12 Hematology and Biochemistry Analysis

The levels of biochemical indexes, including ALT, AST, AKP, CRE, BUN, and UA, were analyzed. Groups of three mice (*n* = 3) were intravenously administered with RT, BNN6, RT+BNN6, PBN, RT+PBN, RT+PBN+αPDL1, PBTN, and RT+PBTN+αPDL1. At 14 days post‐injection, blood samples were collected and centrifuged at 3000 RMP for 5 min to isolate plasma. The plasma was then analyzed using an automated biochemical analyzer to measure the liver and kidney function parameters.

### 4.13 Bioinformatic Analysis

The RNA for gene expression analysis was harvested from the tumor tissues. RNA integrity was assessed using the RNA Nano 6000 Assay Kit on the Bioanalyzer 2100 system (Agilent Technologies, CA, USA). Total RNA was used as input for library preparation. Briefly, mRNA was enriched using poly‐T oligo‐attached magnetic beads and fragmented under elevated temperature. First‐strand cDNA was synthesized with random hexamer primers and M‐MuLV Reverse Transcriptase, followed by second‐strand synthesis using DNA Polymerase I and RNase H. After end repair, 3′adenylation, and adapter ligation, cDNA fragments of 370–420 bp were size‐selected using the AMPure XP system (Beckman Coulter) and amplified by PCR. Library quality was evaluated on the Agilent Bioanalyzer 2100. Clustering was performed on a cBot system using the TruSeq PE Cluster Kit v3‐cBot‐HS (Illumina), and sequencing was conducted on an Illumina NovaSeq platform, generating 150 bp paired‐end reads. Raw RNA‐seq reads were subjected to quality control and adaptor trimming using standard pipelines (FastQC and Trimmomatic). Cleaned reads were aligned to the human reference genome (Rnor_6.0) using HISAT2 (v2.2.1), and gene‐level quantification was performed using featureCounts (Subread v2.0.3). Differential gene expression analysis was conducted with DESeq2 (v1.38.3), and genes with an adjusted *p*‐value < 0.01 and |log2 fold change| > 1 were considered significantly differentially expressed.

For data visualization and downstream analysis, several R packages were employed. Heatmaps were generated using reshaped data matrices via reshape2 (v1.4.4) and customized with ggplot2 (v3.5.2) and ggpubr (v0.6.0). Bar plots and lollipop plots were constructed to depict selected gene expression patterns and pathway enrichment results. We utilized the sparklyr (v1.9.0) and sparklyr.nested (v0.0.4) packages to enhance computational scalability during matrix operations and large‐scale visualization rendering. All figures were rendered in R (v4.4) with unified aesthetics and publication‐quality resolution.

### 4.14 Statistical Analysis

All experiments were conducted with a minimum of two repetitions. The results are presented as mean values ± standard deviation (SD). Randomization was performed for in vivo experiments. Outliers were excluded from the analyses. Statistical analysis for multiple group comparisons was performed using one‐way ANOVA, followed by Tukey's multiple comparison. An unpaired Student's *t*‐test was used for statistical analysis in experiments with two data groups. In the figures, “ns” refers to “no significance”. Survival differences were determined using the log‐rank test. *p‐*values were annotated on the figures. Statistical significance is denoted as *p* < 0.05, *p* < 0.01, *p* < 0.001, and *p* < 0.0001, and ns (not significant) in the figures. The figure legends state the number of animals included in each study.

## Author Contributions

This study was conceived by Fuxin Xue and Linlin Liu. The manuscript was written by Wanze Zhang, Xiaoyan Yin, and Ting wang. Data collation and analysis were performed by Wanze Zhang, Hongfu Zhao, and Zhipeng Zhao. Genomics and transcriptomics analyses were performed by Cheng Tao and Ting Wang. Project administration was conducted by Jinbao Wang and Xuanchu Ge. An investigation was conducted by Yanze Li. Funding acquisitions were provided by Fuxin Xue and Wanze Zhang. All authors contributed to the final version.

## Funding

This work was supported by the Science and Technology Development Program of Jilin Province (YDZJ202601ZYTS632), the Young Talent Program of China‐Japan Union Hospital of Jilin University (2025QM01), Jilin Provincial Department of Science and Technology General Project (YDZJ202501ZYTS075), and Jilin University Bethune Project (2025B14).

## Conflicts of Interest

The authors declare no conflicts of interest.

## Supporting information




**Supporting File**: advs75431‐sup‐0001‐SuppMat.docx.

## Data Availability

Research data are not shared.
