# Supplementary material for: A Radioresistant‐Tumor‐Targeted Nanoparticle for X‐Ray‐Controlled Nitric Oxide Release to Potentiate Radiotherapy
Source: Adv Sci (Weinh). 2026 Apr 22;13(40):e18233. doi: 10.1002/advs.202518233 (PMC13335605; doi:10.1002/advs.202518233)
Supplement: Supplementary file 1 — Supporting File: advs75431‐sup‐0001‐SuppMat.docx. [file ADVS-13-e18233-s001.docx]

Supporting Information

**A Radioresistant-Tumor-Targeted Nanoparticle for X-ray-Controlled Nitric Oxide Release to Potentiate Radiotherapy**

*Wanze Zhang^1#^, Xiaoyan Yin^3#^, Ting Wang^2#^, Hongfu Zhao^1^, Zhipeng Zhao^1^, Jinbao Wang^1^, Cheng Tao^4^, Xuanchu Ge^4^, Yanze Li^4*^, Linlin Liu^1^, Fuxin Xue^1*^*

Wanze Zhang, Hongfu Zhao, Zhipeng Zhao, Jinbao Wang, Linlin Liu, Fuxin Xue

Department of Radiation Oncology, China-Japan Union Hospital of Jilin University, Changchun, Jilin, 130033, China

Ting Wang

Department of Pathology, China-Japan Union Hospital of Jilin University, Changchun, Jilin, 130033, China

Xiaoyan Yin

Department of Radiation Oncology, National Cancer Center/National Clinical Research Center for Cancer/Cancer Hospital, Chinese Academy of Medical Sciences and Peking Union Medical College, Beijing, 100021, China

Cheng Tao, Xuanchu Ge, Yanze Li

Department of Radiation Oncology Physics and Technology, Shandong Cancer Hospital and Institute, Shandong First Medical University and Shandong Academy of Medical Sciences, Jinan, 250117, China

* Corresponding authors.

E-mails: Yanze Li (yanzeli95@gmail.com); Fuxin Xue [(xuefx@jlu.edu.cn)](mailto:(xuefx@jlu.edu.cn))


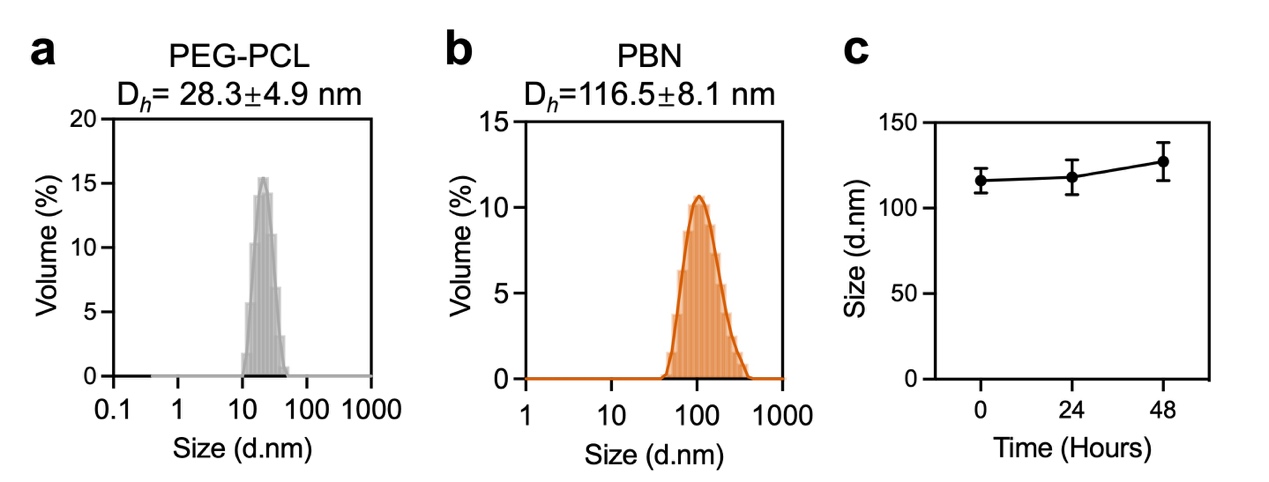


**Figure S1.** DLS of (a) PEG-PCL and (b) PBN. (c) DLS of PBTN incubated in PBS (pH 7.4) at 37 °C for 48 hours (n = 3)


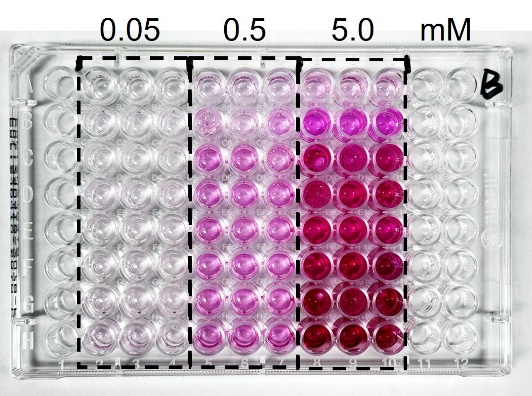


**Figure S2.** NO release amounts of PBTN at different concentrations (0.05-5.0 mM) after irradiation (n = 3).

**
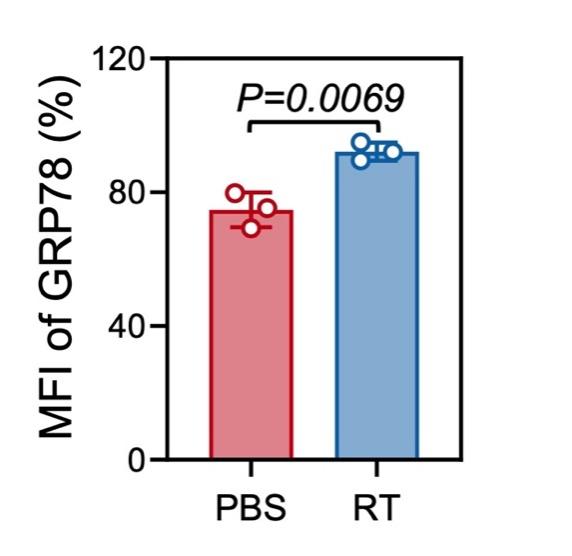
**

**Figure S3.** GRP78 expression of CT26 cells after treatment with PBS and RT (n=3).

**Table S1.** The cytotoxicity of PBN and PBTN against CT26 and L929 cells.

| Cell line | Treatment | IC_50_ (μg/mL) |
| --- | --- | --- |
| CT26 ​ | PBN | 26.32 |
|  | PBN+IR | 3.38 |
|  | PBTN | 10.11 |
|  | PBTN + IR | 0.40 |
| L929 | PBN | > 100 |
|  | PBN + IR | 36.52 |
|  | PBTN | > 100 |
|  | PBTN + IR | 24.88 |


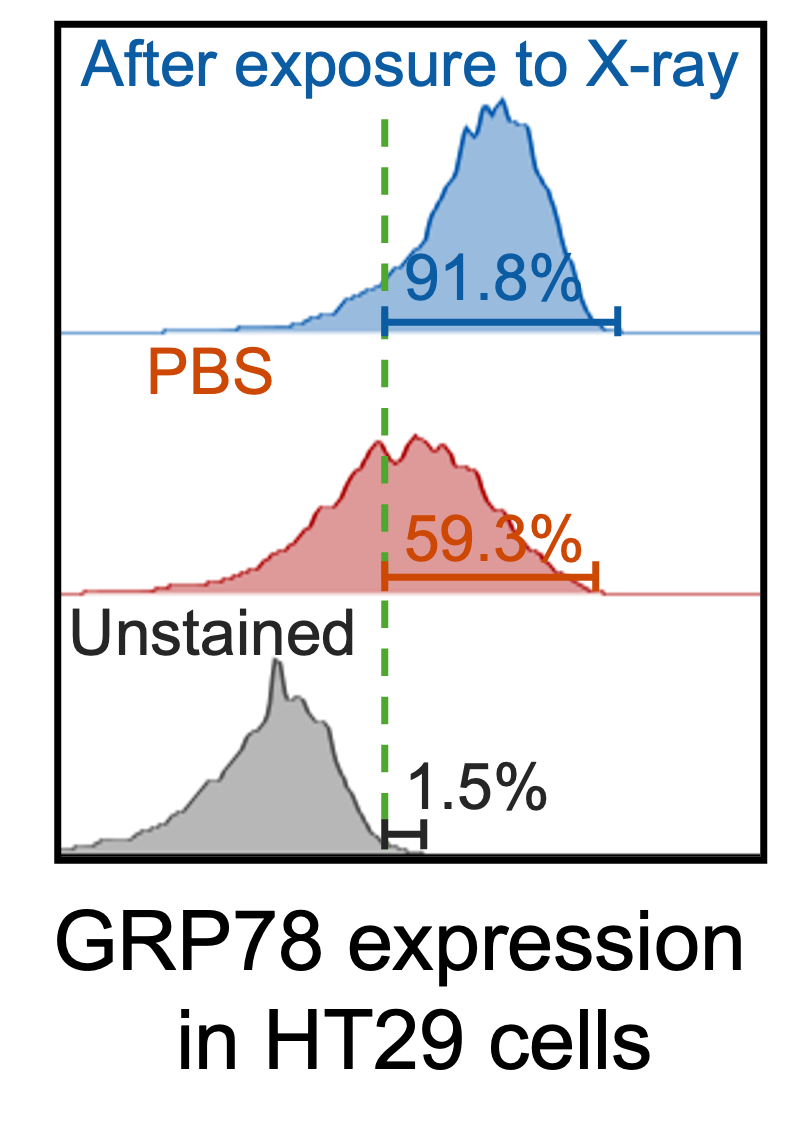


**Figure S4.** GRT78 expression in HT29 cells before and after exposure to X-rays.

**
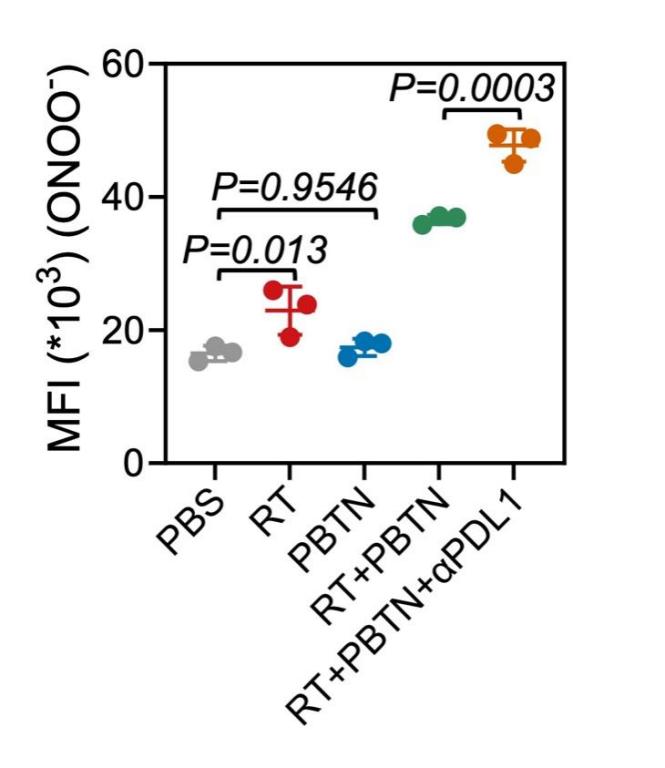
Figure S5.** MFI of ONOO^-^ in tumors of mice after different treatments with PBS, RT, PBTN, RT+PBTN and RT+PBTN+αPDL1 (n=3).

**
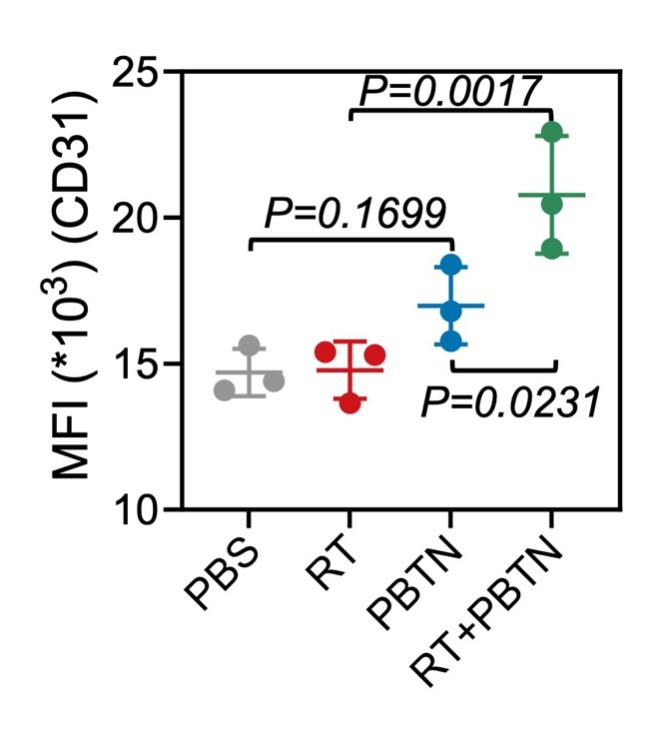
**

**Figure S6.** MFI of CD31 in tumors of mice after different treatments with PBS, RT, PBTN and RT+PBTN (n=3).

**
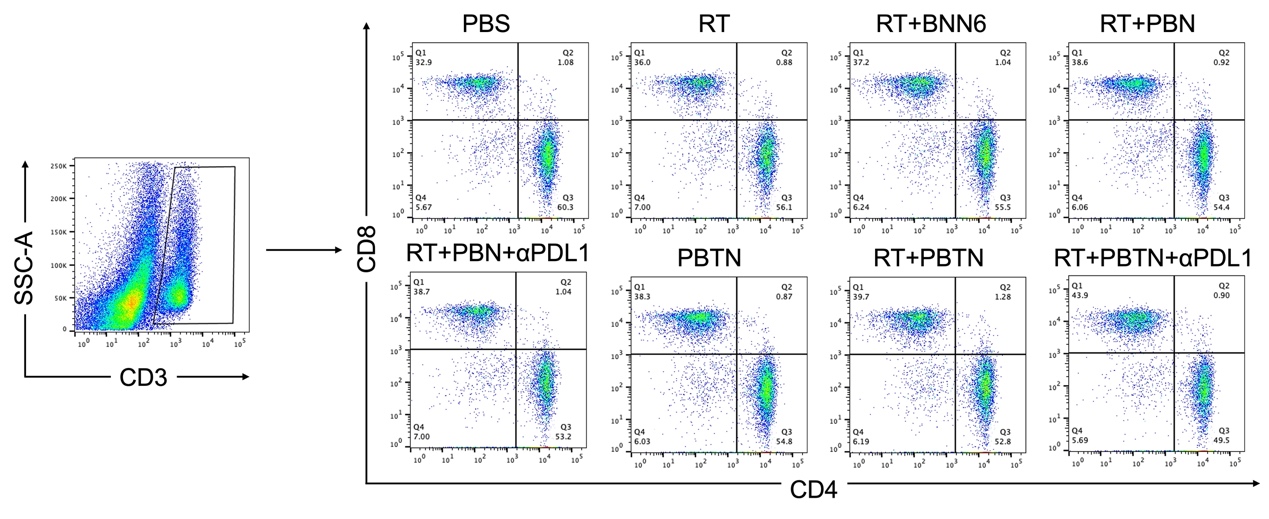
**

**Figure S7.** Flow cytometry of CD3^+^CD4^+^ T cells and CD3^+^CD8^+^ T cells sorting spleens in mice from different treatment groups (PBS, RT, RT+BNN6, RT+PBN, RT+PBN+αPDL1, PBTN, RT+PBTN, RT+PBTN+αPDL1).

**
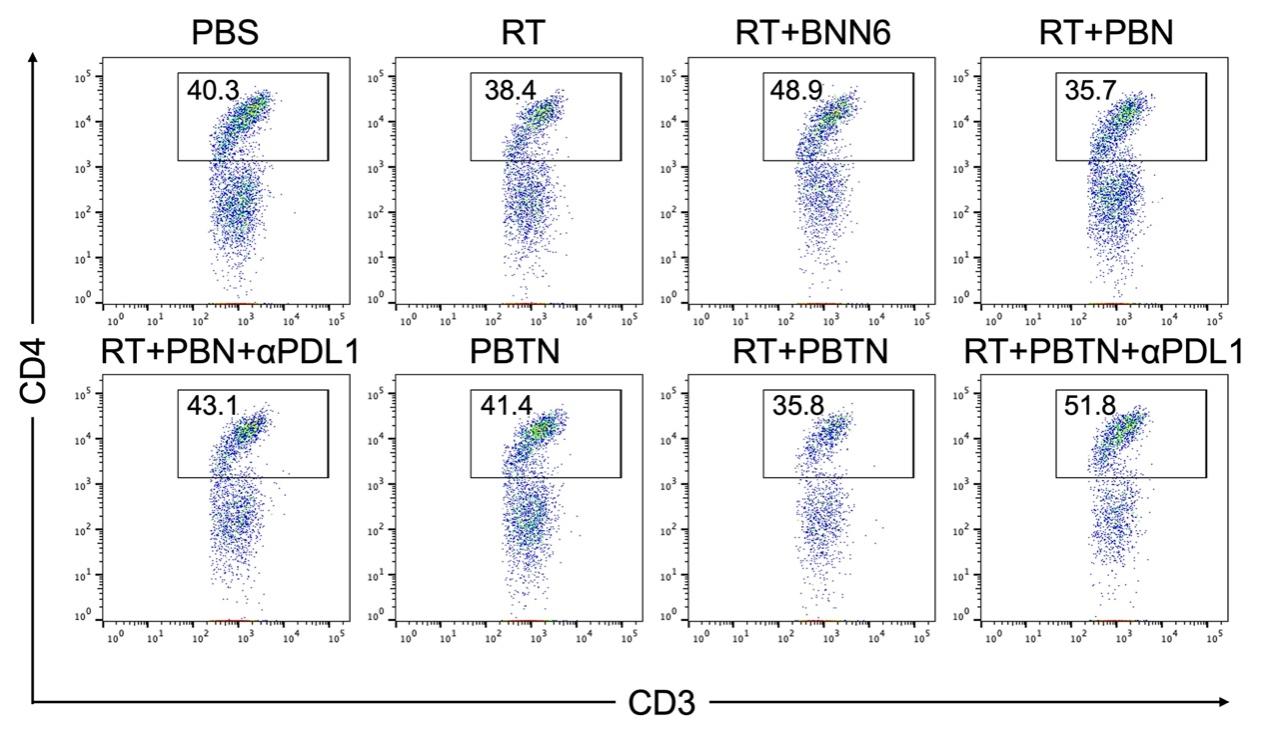
**

**Figure S8.** Flow cytometry of CD3^+^CD4^+^ T cells sorting tumors in mice from different treatment groups (PBS, RT, RT+BNN6, RT+PBN, RT+PBN+αPDL1, PBTN, RT+PBTN, RT+PBTN+αPDL1).

**
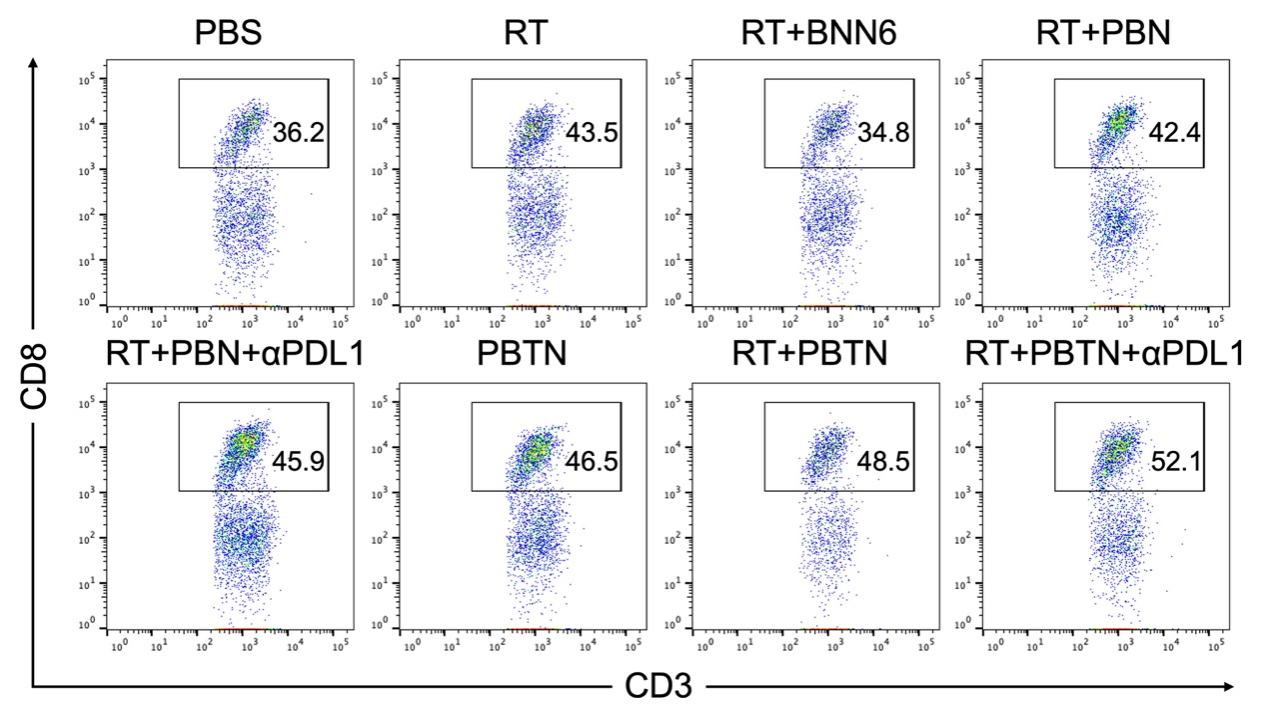
**

**Figure S9.** Flow cytometry of CD3^+^/CD8^+^ T cells sorting tumor in mice from different treatment groups (PBS, RT, RT+BNN6, RT+PBN, RT+PBN+αPDL1, PBTN, RT+PBTN, RT+PBTN+αPDL1).


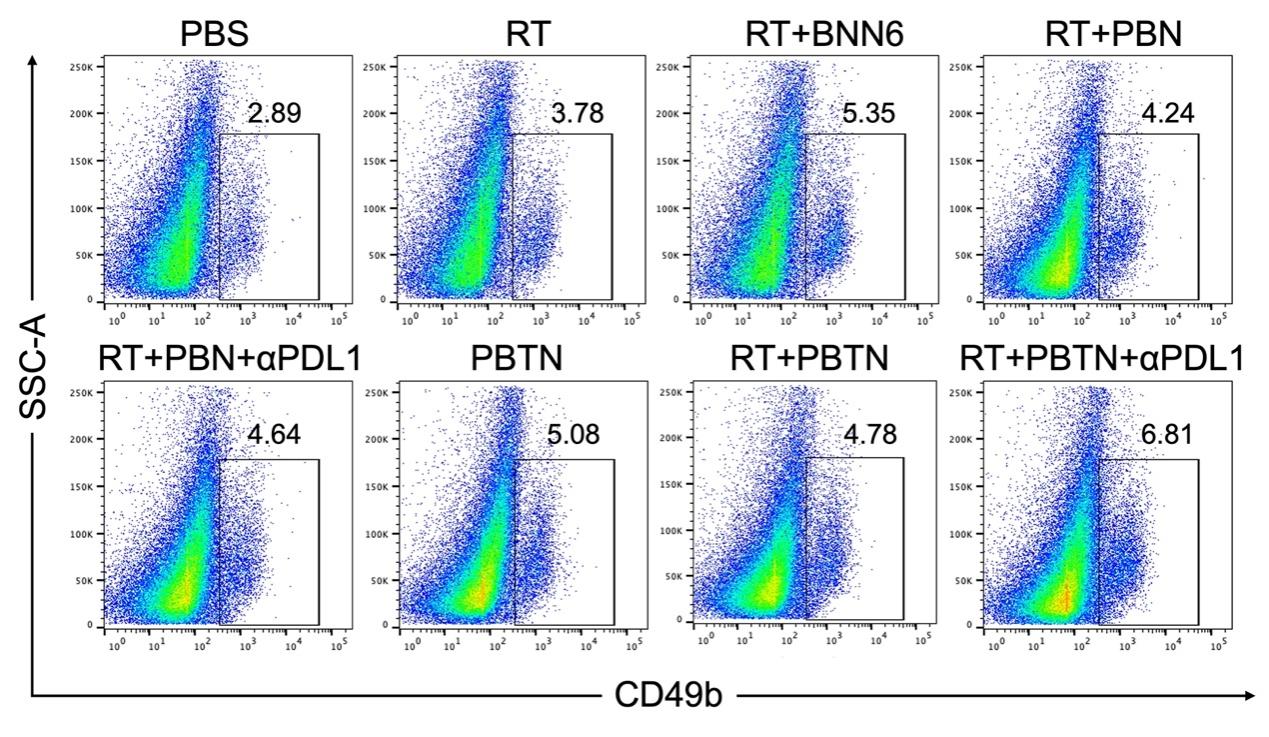


**Figure S10.** Flow cytometry of NK cells sorting tumors in mice from different treatment groups (PBS, RT, RT+BNN6, RT+PBN, RT+PBN+αPDL1, PBTN, RT+PBTN, RT+PBTN+αPDL1).


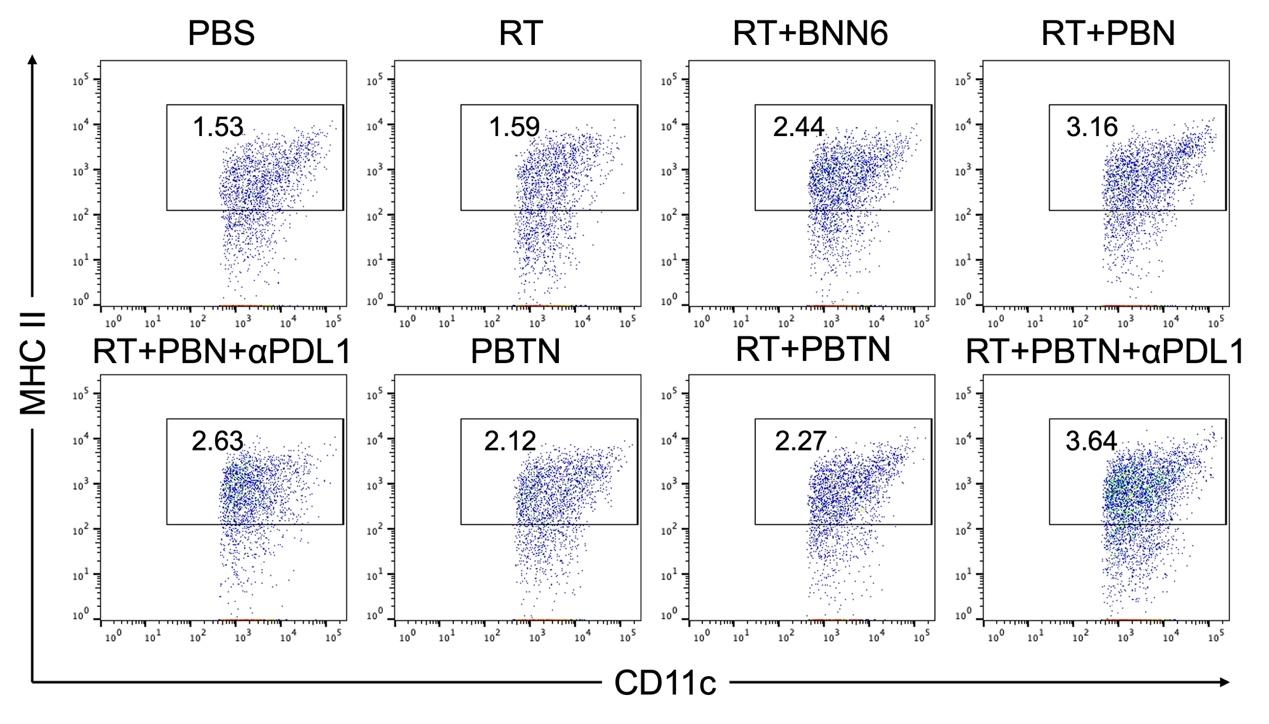


**Figure S11.** Flow cytometry of DCs sorting tumors in mice after different treatments (PBS, RT, RT+BNN6, RT+PBN, RT+PBN+αPDL1, PBTN, RT+PBTN, RT+PBTN+αPDL1).

**
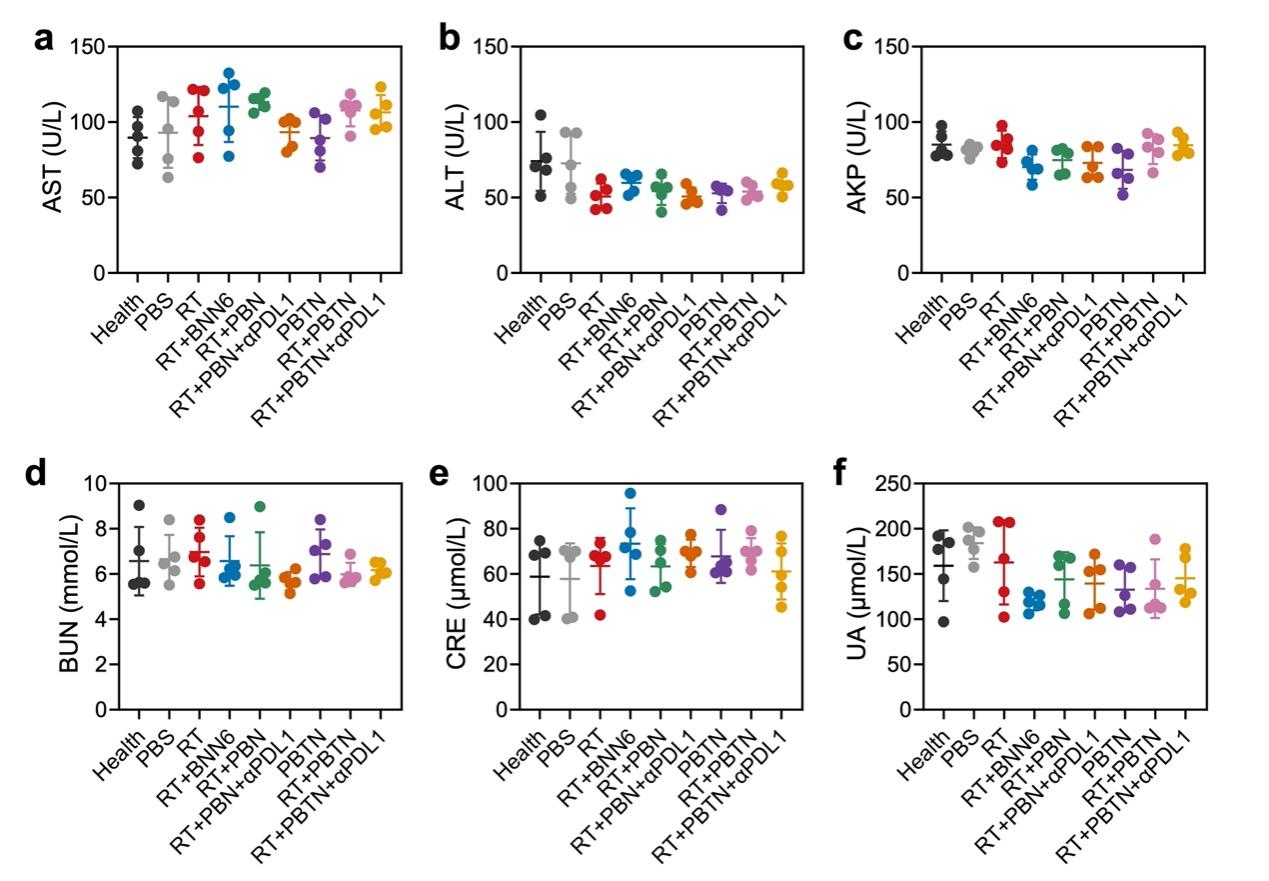
**

**Figure S12.** Serum enzyme contents (AST, ALT, AKP, BUN, CRE and UA) in tumors of mice after different treatments with PBS, RT, RT+BNN6, RT+PBN, RT+PBN+αPDL1, PBTN, RT+PBTN, RT+PBTN+αPDL1 (n = 5).


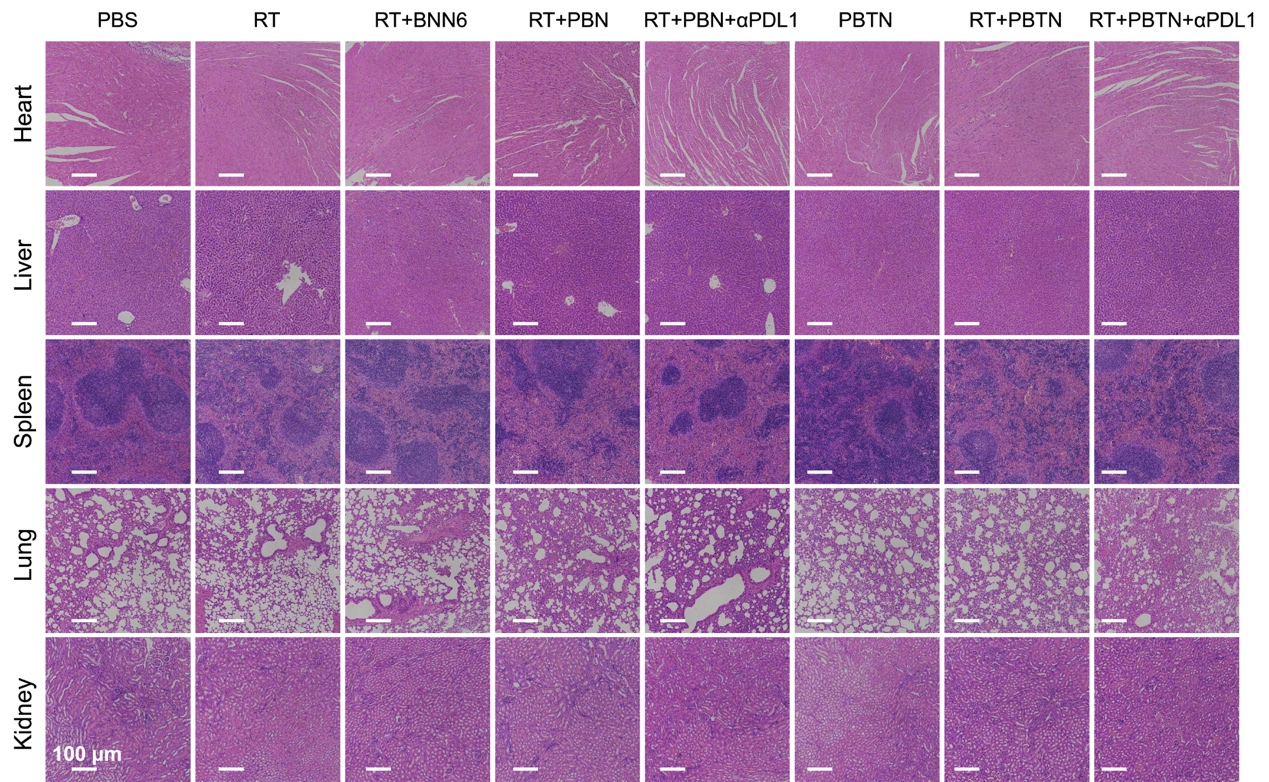


**Figure S13.** H&E images of organs in mice after different treatments PBS, RT, RT+BNN6, RT+PBN, RT+PBN+αPDL1, PBTN, RT+PBTN, RT+PBTN+αPDL1 (Scale bars = 100 μm).

**Table S2.** List of abbreviations in manuscript.

| Abbreviations | Full name​ |
| --- | --- |
| NO | Nitric oxide |
| ONOO^-^ | Peroxynitrite |
| PBN | PEG-PCL-BNN6 nanoparticles |
| PBTN | PEG-PCL-BNN6-GRP78-targeted nanoparticles |
| BNN6 | N,N’-di-sec-butyl-N,N’-dinitroso-1,4-phenylenediamine |
| RT | Radiotherapy |
| ROS | Reactive oxygen species |
| ICD | Immunogenic cell death |
| ISDN | Isosorbide dinitrate |
| ISMN | Isosorbide Mononitrate |
| 2-NML | 2-Nitroimidazole |
| PBS | Phosphate-Buffered Saline |
| FBS | Fetal Bovine Serum |
| TEM | Transmission electron microscope |
| H&E | Hematoxylin and eosin |
| TUNEL | TdT-mediated dUTP nick-end labeling |
| DAPI | 4’,6-diamidino-2-phenylindole |
| AST | Aspartate transaminase |
| ALT | Alanine transaminase |
| AKP | Alkaline phosphatase |
| BUN | Blood urea nitrogen |
| CRE | Creatinine |
| UA | Urea |
| DMF | N,N-Dimethylformamide |
| SSD | Source-to-surface distance |

**Table S3.** The antibodies indicating suppliers and titers in manuscript.

| Antibody | Item Number | | Indicating suppliers | Titers |
| --- | --- | --- | --- | --- |
| FITC anti-mouse CD3ε antibody | 100204 | BioLegend | | 1:200​ |
| APC/Cyanine 7 Anti-Mouse MHCII Antibody | 107630 | BioLegend | | 1:200​ |
| APC anti-mouse CD80 antibody | 104718 | BioLegend | | 1:200 |
| APC anti-mouse CD8 antibody | 100712 | BioLegend | | 1:200​ |
| APC anti-mouse CD4 antibody | 100422 | BioLegend | | 1:200​ |
| PE anti-mouse CD4 antibody | 105014 | BioLegend | | 1:200​ |
| PE anti-mouse CD45 antibody | 103106 | BioLegend | | 1:200​ |
| CD31 (D8V9E) monoclonal antibody | 77699 | **Cell Signaling Technology** | | 1:200 |
| InVivoMab anti-mouse PD-L1 (B7-H1) | BE0101 | **Bio X Cell** | | 1:5 |
